# Automatic intraoperative optical coherence tomography positioning

**DOI:** 10.1007/s11548-020-02135-w

**Published:** 2020-04-02

**Authors:** Matthias Grimm, Hessam Roodaki, Abouzar Eslami, Nassir Navab

**Affiliations:** 1 Technical University of Munich, Garching bei München, Germany; 2 Translational Research Lab, Carl Zeiss Meditec, München, Germany; 3 Johns Hopkins University, Baltimore, USA

**Keywords:** Automatic positioning, Intraoperative optical coherence tomography, Computer-aided ophthalmic surgery

## Abstract

**Purpose:**

Intraoperative optical coherence tomography (iOCT) was recently introduced as a new modality for ophthalmic surgeries. It provides real-time cross-sectional information at a very high resolution. However, properly positioning the scan location during surgery is cumbersome and time-consuming, as a surgeon needs both his hands for surgery. The goal of the present study is to present a method to automatically position an iOCT scan on an anatomy of interest in the context of anterior segment surgeries.

**Methods:**

First, a voice recognition algorithm using a context-free grammar is used to obtain the desired pose from the surgeon. Then, the limbus circle is detected in the microscope image and the iOCT scan is placed accordingly in the *X*–*Y* plane. Next, an iOCT sweep in *Z* direction is conducted and the scan is placed to centre the topmost structure. Finally, the position is fine-tuned using semantic segmentation and a rule-based system.

**Results:**

The logic to position the scan location on various anatomies was evaluated on ex vivo porcine eyes (10 eyes for corneal apex and 7 eyes for cornea, sclera and iris). The mean euclidean distances (± standard deviation) was 76.7 (± 59.2) pixels and 0.298 (± 0.229) mm. The mean execution time (± standard deviation) in seconds for the four anatomies was 15 (± 1.2). The scans have a size of 1024 by 1024 pixels. The method was implemented on a Carl Zeiss OPMI LUMERA 700 with RESCAN 700.

**Conclusion:**

The present study introduces a method to fully automatically position an iOCT scanner. Providing the possibility of changing the OCT scan location via voice commands removes the burden of manual device manipulation from surgeons. This in turn allows them to keep their focus on the surgical task at hand and therefore increase the acceptance of iOCT in the operating room.

## Introduction

Recently, intraoperative optical coherence tomography (iOCT) has been introduced as a new modality to assist eye surgeons during ophthalmic surgery. It provides real-time cross-sectional information at the required high resolution. Furthermore, iOCT is non-invasive and can be coupled with existing operating microscopes. This allows for safer treatment and better outcomes for surgeries in both the anterior and posterior segments of the eye. Several studies have pointed out the potential clinical impact of iOCT for various anterior segment surgeries, especially for glaucoma and cornea surgeries [2, 4]. Despite these possibilities, the acceptance of iOCT in current clinical practice remains low. One of the reasons is the difficulty in interacting with the scanner during procedures. The surgeon needs both his hands for the surgery. Hence, the only options to interact with the scanner are via foot control pedals or having an additional staff member to operate the scanner from a control screen. The second option would require additional staff in the operating room, whereas the first option is cumbersome and results in a steep learning curve.

Properly adjusting the acquisition position of a scanner is the first step required in order to utilize it to its full potential during a surgery. It is also the first step required for most algorithms in the domain of computer-aided interventions. However, it receives very little attention in the literature. For some scanners, such as magnetic resonance imaging or computed tomography, this is understandable as their omnidirectional nature renders the problem superfluous. For other domains, such as freehand ultrasound, this problem is necessarily delegated to the operator. However, even many robotic ultrasound applications require an initial manual positioning. Automizing the positioning step would enable a new generation of algorithms with an unprecedented level of autonomy. This can help make healthcare systems more future-proof, as steps currently executed by additional staff can be autonomously executed by an algorithm. In this work, an automated positioning system is proposed for an intraoperative optical coherence tomography device in the context of the anterior segment (AS) of the eye. The system not only positions the iOCT scan, but also focuses the microscope on the desired location, to obtain the best image quality.

By providing an automated positioning system based on novel deep learning techniques and domain knowledge, and by integrating said positioning system into a high-level application logic using voice control, this paper attempts to overcome the previously mentioned issues. It proposes an intelligent iOCT assistant and therefore a novel interaction paradigm for iOCT. The surgeon issues high-level commands using his voice and the system, powered by artificial intelligence algorithms executes these commands autonomously. This is demonstrated for the example of AS surgeries, but it can be easily extended to other ophthalmic surgery-related applications. The method was tested using a Carl Zeiss OPMI LUMERA 700 with RESCAN 700 (Carl Zeiss Meditec, Germany). Experiments were carried out on ex vivo pig eyes.

## Related work

A method [17] was proposed that deals with the positioning problem from the augmented reality viewpoint. The method helps a technician to properly place a C-arm by visualizing the desired pose in a head-mounted display. However, in this work the desired positions are not automatically computed, but rather manually determined by a technician.

Assuming a proper initial pose, there have been several works on positioning in the context of robotic ultrasound. One study [15] emphasizes the general importance of proper positioning in ultrasound-based applications. Li et al. presented a collaborative robotic ultrasound system with the capability to track kidney stones during breathing [10]. Huang et al. use a depth camera to scan a local plane around the scanning path and obtain the normal direction to set the optimal probe orientation for a robotic ultrasound system [7]. A visual servoing method was proposed to ideally position an ultrasound probe in the in-plane direction in unknown and/or changing environments based on an ultrasound confidence map [1]. Göbl et al. used pre-operative computer tomography scans to determine the best ultrasound probe position and orientation to scan the liver through the rib cage [5]. However, they only calculate a position and do not actually carry out the positioning task. A method conducting a fully autonomous scan of the liver using a robotic ultrasound system was put forward [11]. The method is, however, limited to one specific organ. A feasibility study was conducted in [6]. In this work, desired scan trajectories were marked on a magnetic resonance imaging scan, and then, a depth camera coupled with an ultrasound to magnetic resonance imaging registration was used to guide a robot to move to the patient and autonomously conduct the scans. However, the desired scan trajectories still had to be selected manually.

The most popular network architecture for semantic segmentation is the U-net architecture [12]. It still achieves state-of-the-art performance for a wide variety of medical segmentation tasks today [8]. It was also applied to segmentation in the context of the AS of the eye [13]. The algorithm from [13] is also used as underlying segmentation algorithm for the present work.

Several methods for computer-aided AS surgery have been proposed. One method [14] proposes a complete augmented reality guidance system for big-bubble deep anterior lamellar keratoplasty (DALK) using iOCT. Another group developed a robot [3] for DALK assistance.

## Background knowledge

### Optical coherence tomography

**Fig. 1:**
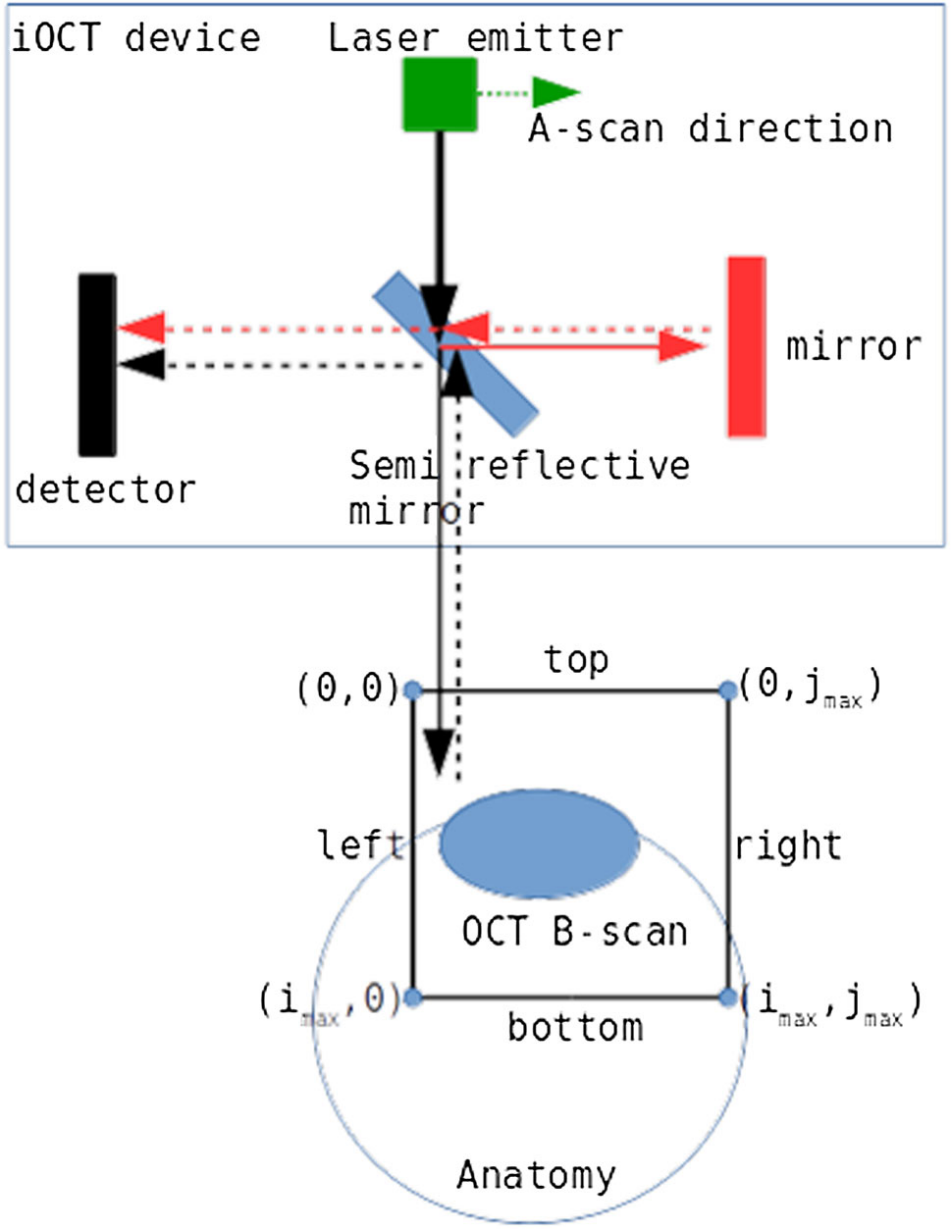
Schematic overview over a spectral domain intraoperative optical coherence tomography device

An iOCT scan *S* is a matrix with *i* rows and *j* columns. The pixel at row *i* and column *j* is denoted as *S*[*i*, *j*]. Row *i* is denoted as *S*[*i*,  : ] and column *j* as *S*[ : , *j*]. The maximum number of rows and columns are denoted as $i_{\max }$ and $j_{\max }$.

An entire scan *S* is called a B-scan. A B-scan consists of several A-scans, namely the columns of *S*.

In this work, a spectral domain OCT is used. It acquires A-scans by emitting laser beams of different wavelengths towards the imaged tissue. The beams pass a semi-reflective mirror, and half of the beams get sent towards the tissue, whereas the other half is sent on a reference path. Both halves rejoin at a detector, where a reconstruction is computed based on the interference pattern. Then the laser emitter is moved to acquire the next A-scan. The imaged depth is controlled by modifying the length of the reference path, by moving a mirror at its end using a so-called reference arm. This is shown in Fig. 1.

The following conventions are used throughout the paper. The top left pixel of a B-scan is the origin in pixel space, with indices increasing towards the bottom right corner. The plane orthogonal to the row direction is denoted as *X*–*Y* plane, whereas the row direction is denoted as *Z* direction. The direction towards decreasing column indices is denoted as left direction, whereas the direction towards increasing column indices is denoted as right direction. Similar, the direction towards increasing row indices is denoted as top direction, whereas the direction towards decreasing row indices is denoted as bottom direction. iOCT scans contain a large amount of speckle noise due to forward and backward scattering of the laser directed towards the anatomy. Currently available iOCT devices are integrated into surgical microscopes, as both devices can share the same optical path. By default, the two devices are calibrated together, allowing to compute locations in *X*–*Y* plane based on the microscope image and position the iOCT accordingly. An iOCT has four programmatically controllable degrees of freedom. These are three translation directions and the rotation around *Z* direction.

### Anterior segment anatomy

The AS consists of three main anatomies: the sclera, the iris and the cornea. The iris acts as an aperture for the lens. The cornea, besides refracting light, acts as a transparent shield for the iris and lens. The highest point of the cornea is called the corneal apex. The sclera is the white, outer protective layer of the eye. The border between the cornea and the sclera is called the limbus. Due to its shape, it is also called limbus circle. The part of the cornea close to the limbus is called the peripheral cornea, whereas the centre of the cornea is called the central cornea. The area enclosed by iris and cornea is called the anterior chamber. The anatomical relation between the three anatomies is depicted in Fig. 3a.

### Clinically relevant poses

There are a number of clinically relevant positions in the AS. For DALK, an incision into the peripheral cornea is made with a needle. Then, a tunnel is created by pushing the needle towards the central cornea. As soon as the needle reaches a suitable point, air is injected. iOCT allows to monitor the current depth of the needle in the cornea, to ensure proper treatment. Therefore, two positions are of interest: the peripheral cornea and the central cornea. The central cornea can be approximated by the corneal apex. For cataract surgery, an incision is made close to the limbus to enter the anterior chamber. Therefore, the limbus is one pose of interest. Many glaucoma treatments, such as trabeculectomy or canaloplasty, require operating at the intersection of iris and sclera. For example during trabeculectomy, a tunnel-shaped implant is implanted between the iris and the sclera. In order to proper place the implant and asses its position, positioning on the sclera and the iris is of interest.

**Fig. 2:**
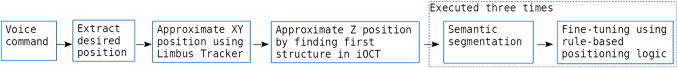
Overview over the proposed method

The proposed method consists of three main steps, which are described below. The desired pose is input via voice commands. Then, the approximate location in the *X*–*Y* plane is determined by detecting the limbus circle in the microscope image. Next, the reference arm of the iOCT is positioned such that the first anatomical structure at the current *X*–*Y* location is in the middle of the B-scan. Next, the position is fine-tuned using a rule-based system and a semantic segmentation of the AS. An overview over all the steps involved is given in Fig. 2.

### Voice commands

**Fig. 3:**
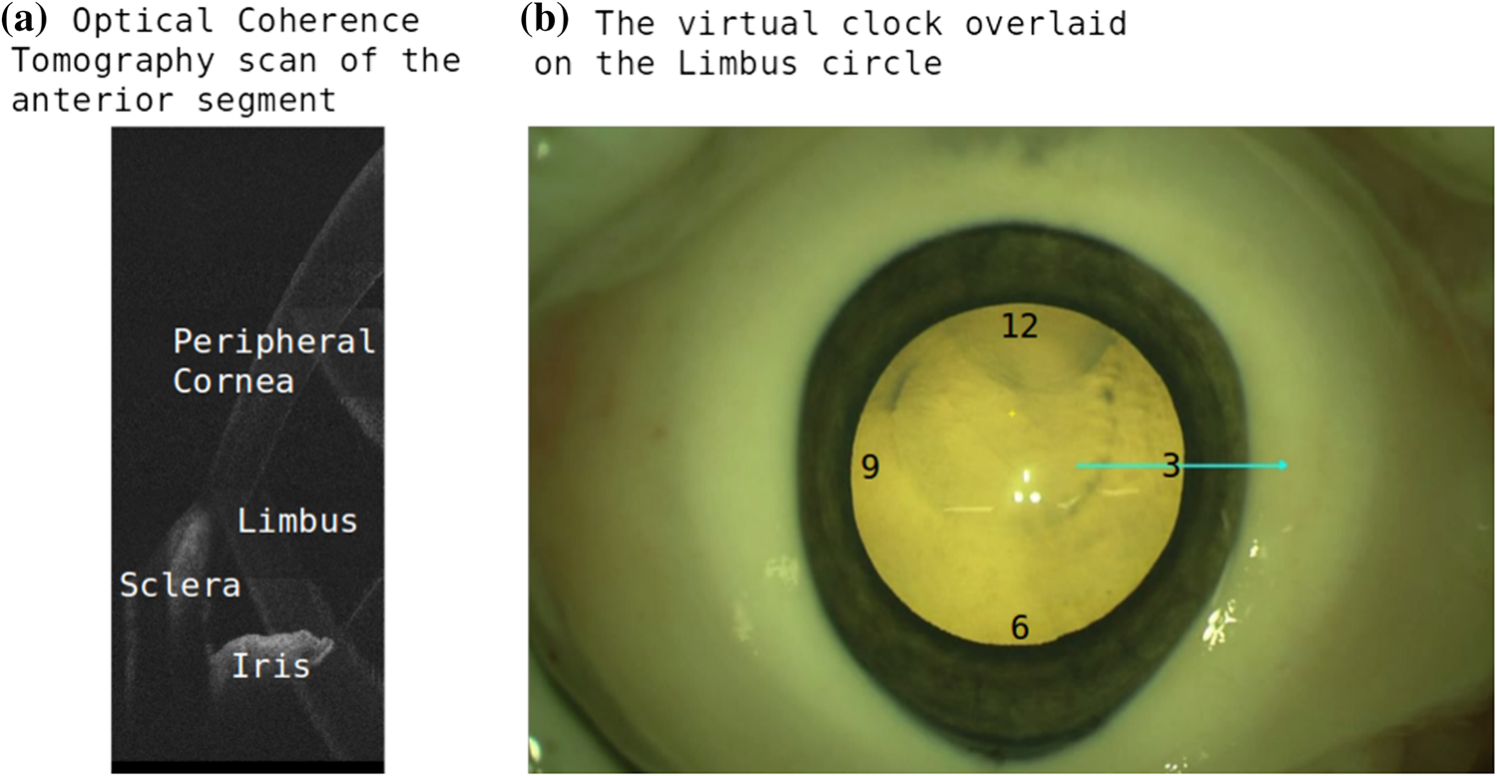
**a** Anatomy of the anterior segment as seen with an iOCT. This image shows multiple scans compounded together for a larger field of view. The central cornea is not visible. The scans were obtained from an ex vivo pig eye; hence, the lens is not properly visible. The contrast of the displayed iOCT image was increased for better visibility. **b** Schematic drawing of the virtual clock superimposed onto the limbus circle. For visualization purposes, only 3, 6, 9 and 12 o’clock are shown. The turquoise arrow indicates the position of the iOCT scan. Hence, the iOCT is placed at 3 o’clock in the image. The image shows an ex vivo pig eye

The surgeon needs both his hands for surgery. Therefore, voice commands are used to obtain the desired pose from him.

The positioning system is able to focus on five anatomies, namely iris, sclera, cornea and apex of the cornea and limbus. The lens was not included, since the system was evaluated on ex vivo pig eyes, where the lens is not properly visible. The apex of the cornea is uniquely defined. All other anatomies span around the entire limbus circle, and hence, there is a multitude of scan locations displaying the target anatomy. From a surgical point of view, these positions are equivalent. However, surgeons have their own preference, as to which side of the limbus they operate on. Therefore, a virtual clock is superimposed onto the limbus circle. Then a position consists of an anatomy and the position on that virtual clock (e.g. 3 o’clock iris). This is shown in Fig. 3b.

This structure is encoded using the following context-free grammar: 
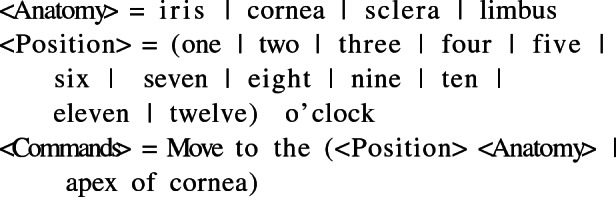
 where | means *or* and $<>$ is used to define a keyword. The voice recognition was implemented using Microsoft Speech API version 4.5.^1^

### Limbus tracking

The first step of the positioning algorithm is to place the iOCT scan at the approximately correct location in the *X*–*Y* plane. The microscope image of the operating microscope is used to find the desired location along the limbus circle. A proprietary limbus tracking algorithm^2^ is used to find the limbus circle in the microscope image. If the limbus circle is not visible, the positioning is terminated, as this indicates that the AS is not imaged. Else, the limbus circle is divided into clock segments as described in “Voice commands” section. Then, if the target anatomy is not the apex of the cornea, the iOCT scan is placed on the corresponding segments. The scan is placed such that the middle of the B-scan is at the limbus circle with the right direction pointing towards the limbus centre. If the target anatomy is the apex of the cornea, the scan location marker is placed at the centre of the limbus circle.

**Fig. 4:**
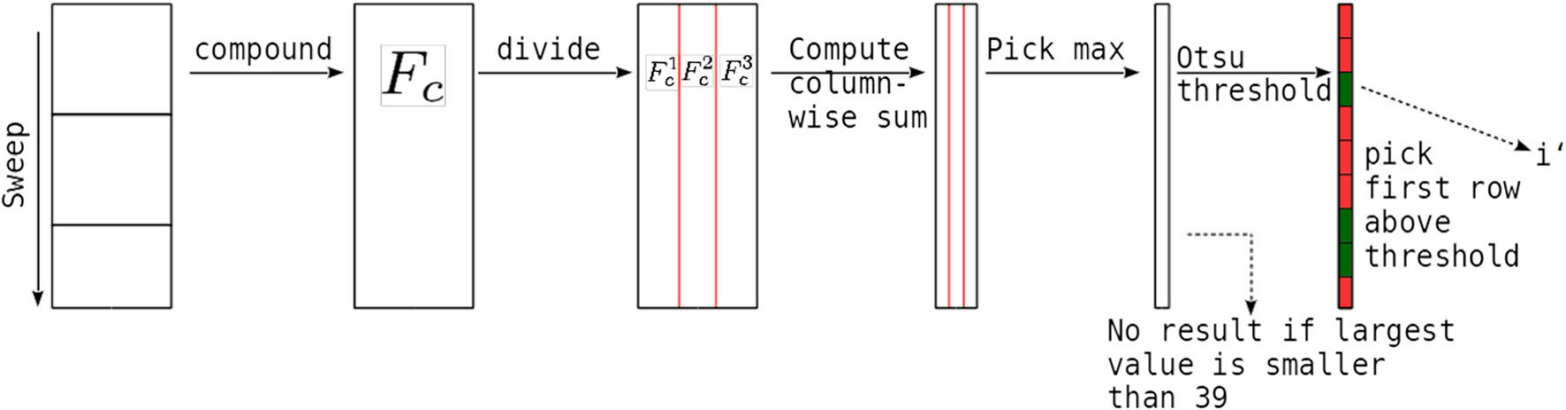
Overview over the algorithm to extract the first row containing structure in the entire OCT reference arm range

### Finding the appropriate location in *Z* direction

The next step is to find the topmost anatomical structure $A_1$ located at the current *X*–*Y* position of the iOCT scan and then subsequently position the reference arm such that $A_1$ appears in the middle row of the resulting B-scan. An overview over the method is depicted in Fig. 4.

First, a compounded frame $F_c$ is created by conducting a sweep over the entire reference arm range of the iOCT device and compounding the individual frames in *Z* direction. In case of an overlap, the overlapped regions are averaged. The goal is to find the topmost row $i'$ of $F_c$ which images a part of $A_1$.

Since not necessarily all columns of $i'$ contain a structure, $F_c$ is partitioned into three equal sized parts $F_{c}^{q}, q = 1,2,3$, where $F_{c}^{q} = F_{c}[:,(q-1) \cdot (j_{\max }/3):q \cdot (j_{\max }/3)]$. Then, each $F_{c}^{q}$ is reduced to one value per row, namely the mean intensity for that row $F_{c}^{q}[i,0] = mean(F_{c}^{q}[i,:]), i = 0 \ldots i_{\max }, q = 1,2,3$. A new frame $F_{c}'$ with one column is created where $F_{c}'[i,0] = \mathrm{max}(F_{c}^{1}[i,0], F_{c}^{2}[i,0], F_{c}^{3}[i,0]), i = 0 \ldots i_{\max }$.

Next, Otsu’s thresholding method [9] is applied to $F_{c}'$. Then $i'$ is assumed to be the first row of $F_{c}'$ whose value is above the Otsu threshold.

1 $$\begin{aligned} i' = \mathrm{arg}\ \mathrm{min}_{i}\ F_{c}'[i,0],\ \mathrm{subject\,to}\ F_{c}'[i,0]\ \ge \ \text {thresh}_\mathrm{otsu} \end{aligned}$$

where ${\text {thresh}}_\mathrm{otsu}$ is the threshold returned by Otsu’s method.

Otsu’s method assumes that the pixel intensities are generated by two distribution: one belonging to background (i.e. noise) and the other one to foreground (i.e. anatomy). The Otsu threshold is then the threshold that achieves the best separation of the two distributions. In iOCT scans, anatomical structures have higher intensity than noise, and hence, the class corresponding to intensities above the Otsu threshold is assumed to contain anatomical structures. If the largest intensity in $F_{c}'$ is lower than an empirically determined value of 39, then no structure can be found for the entire reference arm range. In this case, the algorithm returns without finding a result and the positioning is terminated.

Finally, the microscope head is moved such that $i'$ is as close to the middle of the reference arm range as possible and the reference arm is subsequently moved, such that $i'$ is in the middle of the resulting B-scan. Since the middle of the reference arm range is where the optical focus of the microscope lies, this results in the $A_1$ being in optical focus. Furthermore, the iOCT image quality is better when in optical focus.

### Anterior segment segmentation

After finding the appropriate location in *Z* direction, the iOCT device is positioned at this location. In order to fine-tune the position, a semantic segmentation of the iOCT B-scan is employed. This is based on a previous work [13].

The network architecture is a slight modification of the U-net architecture [12]. The input is an iOCT scan of the AS. The output are probability maps for four classes: cornea, sclera, iris and noise. The input scans are resized to a size of 384 by 384 pixels. The method was developed on ex vivo pig eyes.

### Rule-based positioning

**Fig. 5:**
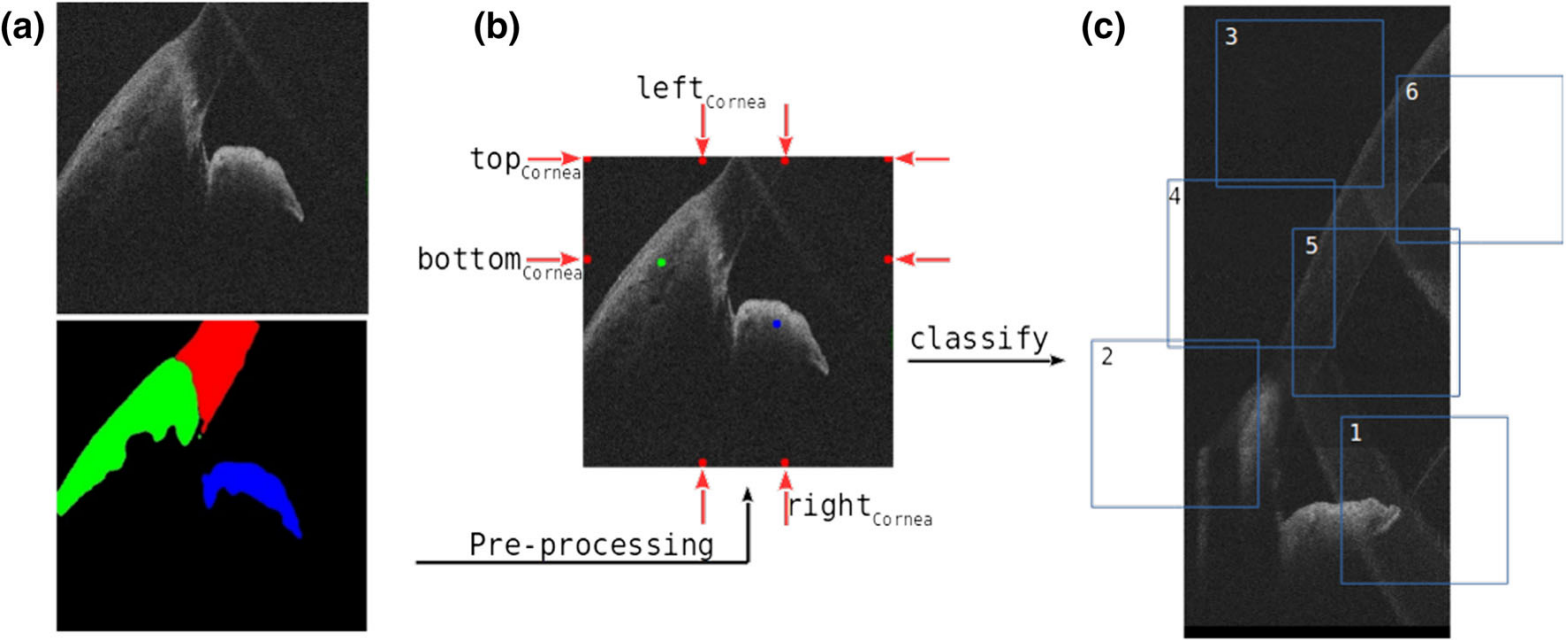
Overview over the rule-based positioning logic if the target is not the apex of cornea. First, the iOCT scan is segmented (**a**). Then a pre-processing step is applied to extract the centroid of the $C_\mathrm{Sclera}$ (green point), the $C_\mathrm{Iris} (blue point)$ and $\mathrm{top}_\mathrm{Cornea}$, $\mathrm{bottom}_\mathrm{Cornea}$, $\mathrm{left}_\mathrm{Cornea}$ and $\mathrm{right}_\mathrm{Cornea}$ (**b**). Next, the position is classified into one of the six classes, based on the extracted features (**c**). Finally, a displacement depending on the class and target anatomy is looked up. The contrast of the displayed iOCT images was increased for better visibility

The final step of the method is a rule-based positioning logic to fine-tune the current location. The positioning logic receives as input the output of the AS segmentation for the classes iris, sclera and cornea. The probability maps are thresholded such that probabilities above 0.5 are mapped to *one* (anatomy) and probabilities below are mapped to *zero* (no anatomy). The output is an offset to the scan location and the reference arm. The logic is Markovian. Three steps are executed, until the final position is reached. Two different algorithms are executed, depending on whether the target anatomy is the apex of the cornea or not.

#### Apex of cornea

The goal is to reach the topmost point of the cornea. First, the scan pattern is changed from single line to cross (i.e. two perpendicular scans who intersect in the middle of the B-scan). The topmost point of the cornea in the corresponding segmentation mask is obtained. Then, the scan location marker is moved such that the point is in the middle of the resulting B-scan. Furthermore, the reference arm is moved such that this point is at the top third of the resulting B-scan. The next acquired B-scan will be the other scan from the cross pattern.

#### Other anatomies

**Fig. 6:**
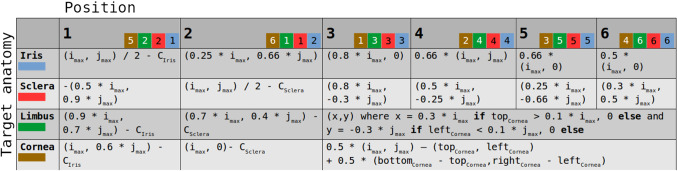
Displacements depending on the target anatomy and the result of the position classification. The displacements are represented by a vector (*i*, *j*), where *i* is the displacement in row direction and *j* is the displacement in column direction. All units are in pixels. The coloured rectangles in the first row indicate in which order the rules for the position classification are evaluated depending on the target anatomy. The different colours represent the different target anatomies: blue for iris, green for sclera, orange for limbus and purple for cornea

There are three steps involved. First, a pre-processing step is applied on the segmentation masks to extract the necessary features. Then, the current position is classified into one of the six classes, based on the previously extracted features. Finally, a displacement is retrieved based on the class and the target anatomy.

***Pre-processing*** The goal of the pre-processing step is to extract meaningful quantities from the segmentation masks for subsequent classification.

First, a contour extraction mechanism [16] is applied to the segmentation masks of the iris and sclera. The centroid of the extracted contours are computed and denoted as $C_\mathrm{Iris}$ and $C_\mathrm{Sclera}$, respectively. If no contour is found, the corresponding values are set to $(-1, -1)$.

Second, the extent of the cornea mask is detected. Therefore, the topmost and bottommost rows and leftmost and rightmost columns containing a cornea denoted as $\mathrm{top}_\mathrm{Cornea}$, $\mathrm{bottom}_\mathrm{Cornea}$, $\mathrm{left}_\mathrm{Cornea}$ and $\mathrm{right}_\mathrm{Cornea}$, respectively. Due to the positioning on the limbus, $\mathrm{left}_\mathrm{Cornea}$ is the column farthest away from the limbus centre. The quantities are extracted as follows. First, a vector *r* is built. *r*[*i*] is *one*, if *I*[*i*,  : ] contains more than eight pixels predicted to be cornea and *zero* else. *I* refers to the iOCT B-scan. Then *r* is partitioned into groups of five successive rows, starting from top. $\mathrm{top}_\mathrm{Cornea}$ is assumed to be the middle row of the first group starting from top that has at least one nonzero entry in *r*. If no such group is found, then $\mathrm{top}_\mathrm{Cornea}$ is set to $-1$. If $\mathrm{top}_\mathrm{Cornea}$ is set to $-1$, then $\mathrm{bottom}_\mathrm{Cornea}$, $\mathrm{left}_\mathrm{Cornea}$ and $\mathrm{right}_\mathrm{Cornea}$ are also set to $-1$.

Else, $\mathrm{bottom}_\mathrm{Cornea}$ is assumed to be the middle of the first group after the group belonging to $\mathrm{top}_\mathrm{Cornea}$ for which the sum of nonzero entries in $r_i$ for the current group and the previous group is less than five. If no such entry is detected, then $\mathrm{bottom}_\mathrm{Cornea}$ is assigned to the last row of the scan.

$\mathrm{left}_\mathrm{Cornea}$ is the leftmost A-scan containing more than five pixels predicted to be cornea. If no such A-scan exists, then $\mathrm{left}_\mathrm{Cornea}$ and $\mathrm{right}_\mathrm{Cornea}$ are set to $-1$.

$\mathrm{right}_\mathrm{Cornea}$ is the rightmost A-scan containing more than five pixels predicted to be cornea.

The partitioning in groups of five is done due to errors in the segmentation for low-quality iOCT scans. In this case, the segmentation yields holes in the predicted cornea. Reducing the granularity of the pre-processing allows for greater robustness against these holes.

***Position classification*** The multitude of potential positions is represented by six representative classes. Examples of these are shown in Fig. 5c. The following set of rules is used to classify the current position into one of the six classes.

- Position 1: $$C_\mathrm{Iris} \ \ne \ (-1, -1) \ \mathrm{and} \ C_\mathrm{Sclera} \ = \ (-1, -1)$$
- Position 2: $$C_\mathrm{Sclera} \ \ne \ (-1, -1) \ \mathrm{and} \ C_\mathrm{Iris} \ = \ (-1, -1)$$
- Position 3: $$\mathrm{bottom}_\mathrm{Cornea} \ < \ 0.9 \cdot i_{\max }$$
- Position 4: $$\mathrm{top}_\mathrm{Cornea} \ > \ 0.8 \cdot i_{\max } \ \mathrm{and} \ \mathrm{bottom}_\mathrm{Cornea} \ \le \ 0.9 \cdot i_{\max }$$
- Position 5: $$\mathrm{left}_\mathrm{Cornea} \ > \ 0.5 \cdot j_{\max } \ \mathrm{and} \ \mathrm{top}_\mathrm{Cornea} \ \le \ 0.8 \ \mathrm{and} \ \mathrm{bottom}_\mathrm{Cornea} \ \le \ 0.9 \cdot i_{\max }$$
- Position 6: $$\mathrm{left}_\mathrm{Cornea} \ < \ 0.1 \cdot j_{\max } \ \mathrm{and} \ \mathrm{top}_\mathrm{Cornea} \ \le \ 0.8 \ \mathrm{and} \ \mathrm{bottom}_\mathrm{Cornea} \ \le \ 0.9 \cdot i_{\max }$$

The rules are not exclusive. Hence, they are checked in different orders depending on the target anatomy. The position is classified according to the first rule that evaluates positively. The evaluation order depending on the target anatomy is shown in Fig. 6.

***Displacements*** The final step of the positioning logic is to look up and execute the displacements corresponding to the position class and the target anatomy. If none of the rules was evaluated positively, then a random displacement is returned. The displacements are shown in Fig. 6. The displacements are computed in pixel units. Then, they are transformed into millimetres and applied as an offset to the program controlling the iOCT.

## Results

The method was evaluated on ex vivo pig eyes. The logic for moving to the apex of the cornea was evaluated on ten pig eyes, whereas the logic to position on the other anatomies was evaluated using seven pig eyes. The starting point was a random location. Before randomly positioning the scan, the microscope head was moved such that the anatomy of interest was in the range of the scanner.

**Fig. 7:**
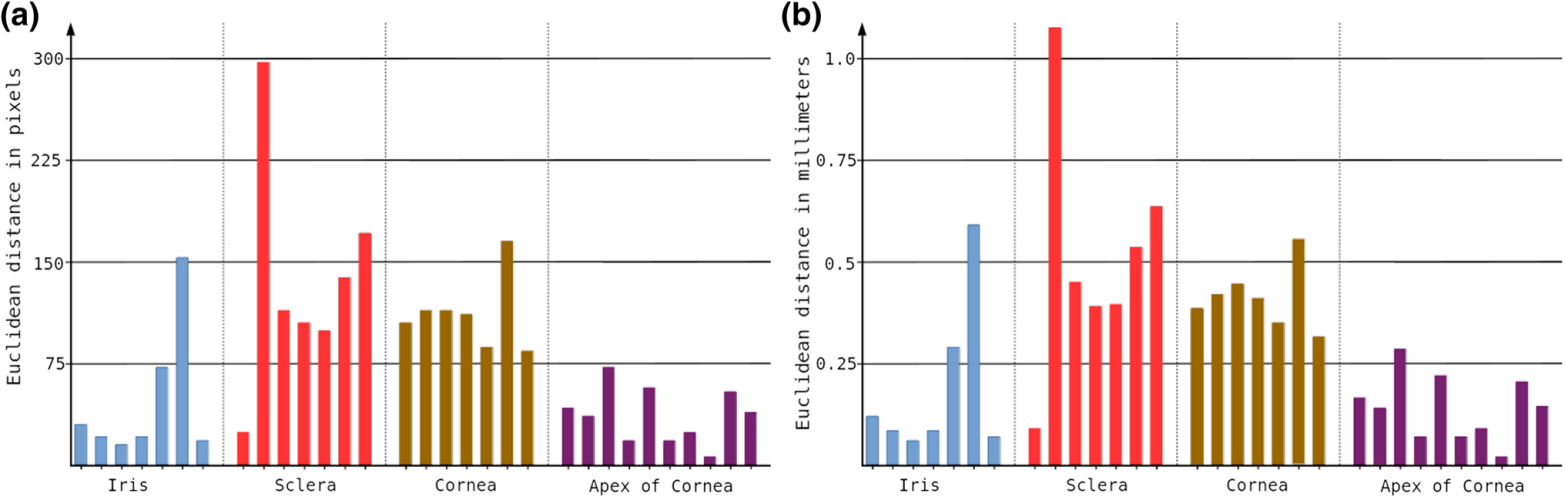
Euclidean distance between the scan location marker returned by the algorithm and the desired scan location marker position in pixels (**a**) and millimetres (**b**) for iris, sclera, cornea and apex of cornea. The slight differences in the relative heights between **a** and **b** are due to the anisotropic resolution of the iOCT scans

**Fig. 8:**
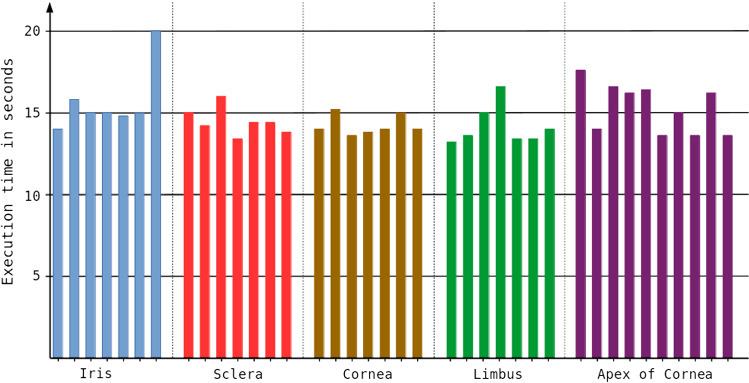
Execution time in seconds for the positioning logic

Ground truths were manually obtained by labelling the apex of the cornea $A_\mathrm{Cornea}'$, and segmenting iris, cornea and sclera. Then, the centroids $C_\mathrm{Iris}'$, $C_\mathrm{Cornea}'$, $C_\mathrm{Sclera}'$ of the ground-truth segmentation masks were calculated. Furthermore, the limbus was manually marked as the line dividing cornea and sclera. Segmentations were done on the final B-scan. The B-scans have a size of 1024 by 1024 pixels and cover 2.8 mm in *Z* and 5 mm in the *X* direction.

The euclidean distance between $C_\mathrm{Iris}'$, $C_\mathrm{Cornea}'$, $C_\mathrm{Sclera}'$, $A_\mathrm{Cornea}'$ and the middle of the scan is depicted in Fig. 7. As can be seen, the distance for sclera and cornea are higher than those for iris and apex. This is because for cornea, the position is not optimized by centring the centroid, but rather based on the $\mathrm{top}_\mathrm{Cornea}$, $\mathrm{left}_\mathrm{Cornea}$, $\mathrm{right}_\mathrm{Cornea}$ and $\mathrm{bottom}_\mathrm{Cornea}$. The error for the sclera is a higher, due to its large extent. The sclera is reasonably centred after three steps of the fine-tuning. However, during each step more parts of the sclera become visible, and hence, the centroid’s location is continuously changing. For the limbus, it is difficult to give a single desired location. Hence, no euclidean error was computed. Instead, it was only evaluated whether the limbus line was in the desired region of the image. Positioning on the limbus is important to prepare an access to the anterior chamber. Therefore, the limbus needs to be in the bottom half of the scan. Furthermore, in order to see the tool approaching, it is necessary that the limbus is located at the right $80\%$ of the scan. This was true for each test case.

The execution time of the positioning logic is shown in Fig. 8. With an average of 15 s, the execution time is the biggest downside of the proposed method.

## Discussion

This paper presented an automated positioning framework for iOCT in the context of AS surgeries. The framework encodes desired poses in a context-free grammar to allow for a natural voice interface. Then, classic computer vision techniques are utilized to find an approximate position. Finally, a rule-based system operating on the output of a semantic segmentation is used to obtain the final position.

The method can be plugged before existing algorithms, allowing for a new level of autonomy. Furthermore, the proposed paradigm of combining voice recognition with contextual artificial intelligence has the potential to improve the acceptance of iOCT in clinical practice. The main downside of the proposed method is the execution time. With an average of 15 s, it is too high for constant use throughout a surgery. However, it is still acceptable for initial positioning during the start of a surgery and occasional repositioning during new phases of a surgery.

A considerable portion of the long execution time is due to the limitation in speed of stepper motors in the system. These motors are responsible for the movement of the reference arm and the microscope head in current microscopes equipped with OCT. Since the movement of these stepper motors are not synchronized with the internal interferometer, acquiring OCT B-scans while the reference arm is moving introduces artefacts that affect computer vision algorithms negatively. In order to guarantee artefact-free frames, the next scan after a completed motion needs to be skipped, effectively halving the frame rate. During the initial search to find the approximate *Z* location, a large frame is assembled covering the entire 35 mm range of the reference arm. This requires constant starting and stopping of the motors and skipping frames. The motor movements associated with this phase alone take between 5.2 and 6.5 s depending on the starting position. The movement required to place a detected structure in the optical focus takes up to 3.8 s. Hence, an applicable solution to reduce the execution time is to use faster motors or alternative optics. Several algorithmic improvements can also be incorporated to reduce the required mechanical movements. One solution is to limit the search range when looking for an initial *Z* location. The initial execution of the method could be done using the full reference arm range, whereas subsequent executions could use a reduced range as the scanner would already be approximately positioned. Another solution is to change the logic for finding an approximate *Z* position. The current logic builds a large frame covering the entire reference arm range and then finds a location in this frame. Alternatively, a method could be developed that works frame by frame. As soon as a structure is found, the method is terminated and that structure is used in subsequent computations. The method was only evaluated on pig eyes; however, the anatomy of pig eyes and human eyes is very similar. Future work could include extending the proposed method to other modalities, such as robotic ultrasound.

To conclude, the present approach releases eye surgeons from the burden of manually positioning the iOCT scanner, thereby allowing them to place more focus on more important aspects of their surgeries.

## Footnotes

1 Microsoft Corporation, Redmond, Washington, USA.

2 Carl Zeiss Meditec, Germany.
